# Relationships of interleukin-10 with the regulatory T cell ratio and prognosis of cervical cancer patients

**DOI:** 10.6061/clinics/2018/e679

**Published:** 2018-11-23

**Authors:** Beibei Wang, Haoyu Wang, Peiquan Li, Liangliang Wang, Hongli Liu, Jingbo Liu, Lihua Wang

**Affiliations:** I Department of gynecologic oncology, the First Affiliated Hospital of Bengbu Medical College, Anhui, China; II Department of orthopaedics, the Third People‘s Hospital of Bengbu, Anhui, China; III Department of gynecologic oncology, Renji Affiliated Hospital of Shanghai Jiao Tong University, Shanghai, China

**Keywords:** Interleukin-10, Regulatory T Cells, Cervical Cancer, ELISA, Prognosis

## Abstract

**OBJECTIVE:**

This study investigated serum interleukin-10 (IL-10) levels, changes in peripheral blood CD4+CD25+ regulatory T cell (PBCDT) ratios, and the prognosis of cervical cancer (CC) patients.

**METHODS:**

Seventy patients with CC composed the observation group, and 70 healthy subjects composed the control group. The PBCDT ratios in the CC patients and healthy subjects were calculated. Serum IL-10 levels were detected with a double antibody sandwich enzyme-linked immunosorbent assay (ELISA).

**RESULTS:**

The PBCDT ratio was higher in the patients with active CC [12.16±2.41%] than in the control subjects [6.34±1.05%]. Serum IL-10 levels were higher in the patients with CC [384±106 pg/ml] than in the control subjects [104±50 pg/ml]; the differences in both PBCDT ratio and IL-10 level were statistically significant (*p*<0.01). Serum IL-10 levels were positively correlated with PBCDT ratios (r=0.375, *p*<0.05). The 5-year patient survival rate was significantly higher in the low serum IL-10 group (64.2%) than in the high serum IL-10 group (42.8%, *p*=0.012).

**CONCLUSIONS:**

PBCDT ratios and serum IL-10 levels are related to CC activity. These factors are reciprocally related and influence one another, and both are involved in the development and progression of CC. Low IL-10 expression is beneficial regarding the survival of patients with CC.

## INTRODUCTION

In the 21^st^ century, there is a high incidence of cancer in women. As one common malignant tumor in women, cervical cancer (CC) ranks third in terms of incidence among all malignant tumor types 1. In many developed countries, cancer is one of the major causes of death in women. Approximately 130,000 people are newly diagnosed with CC in China each year, accounting for approximately 25% of all of the CC patients in the world 2. CC is a long-term, reversible disease. If treatment is provided in the early stages of CC, patients have a 5-year survival rate of up to 90% 3. In many countries, the incidence of CC has significantly decreased. In developed countries, the mortality of CC has dropped significantly by more than 50%, mainly due to the diagnosis and treatment of early-stage CC 4. In recent years, the human papillomavirus (HPV) infection rate in China has increased every year, mainly due to unprotected sexual intercourse and changes in the social lives of patients. As a result, the incidence of CC is also rising 5. According to the statistics, the global age of onset of CC has decreased from 60 years in the 1950s to 50 years in the 1990s. Therefore, the treatment and prevention of CC are public health problems 6.

A previous study 7 found that a large number of regulatory T (Treg) cells, mainly CD4+CD25+ Treg cells, infiltrate the tissue around tumors. It was reported that the CD4+CD25+ Treg cell population is generally increased in the peripheral blood and tumor tissue of patients with stomach cancer, lung cancer, liver cancer, colon cancer, melanoma, and other types of cancer. The CD4+CD25+ Treg cell count is negatively correlated with tumor development and prognosis 8-12. CD4+CD25+ Treg cells have been proposed to maintain the body's immune tolerance and prevent an immune response to homologous tumor cells, thereby playing a crucial role in tumor development. As an important member of the interleukin (IL) family, IL-10 is a multifunctional cytokine that regulates cell growth and differentiation and participates in inflammatory and immune responses 13. CD4+CD25+ Treg cells and CD4+CD25- effector T (Teff) cells jointly regulate the secretion of IL-10, TGF-β, and other factors and play immunosuppressive roles by binding with and secreting IL-10 14. Therefore, this study investigated the changes in human peripheral blood CD4+CD25+ Treg cell ratios and serum IL-10 levels and their relationships with CC patient prognosis.

## MATERIALS AND METHODS

### Clinical information

Seventy patients with CC, confirmed by pathologists via biopsy, in our hospital were assigned to the observation group. Their characteristics were as follows: age: 36-84 years; mean age: 50.5±11.61 years; clinical staging: stage I: n=9, stage II: n=45, and stage III: n=16; and histological type: adenocarcinoma: n=3, squamous carcinoma: n=66, and adenosquamous carcinoma: n=1. Seventy healthy subjects at the physical examination center of our hospital were assigned to the control group. Their characteristics were as follows: age: 35-70 years; and mean age: 48.8±9.5 years. The study was approved by the ethics committee of our hospital. All of the subjects or their families signed the informed consent form. No significant differences in gender, age, or any other clinical characteristics were found between the two groups (Table 1).

The inclusion criteria were as follows: confirmed diagnosis of CC; no blood relationship with other study patients; no liver cirrhosis, autoimmune disease, respiratory disease, depression, hypertension, or diabetes; no serious infections; and no recent blood transfusions.

The exclusion criteria were as follows: course of disease <1 year; recent drug treatment; other genetic diseases; no history of radiotherapy or chemotherapy; autism; memory disorders; hearing disorders; noncompliance with follow-up; or incomplete clinical information.

### Reagents

A flow cytometry Treg Cell Assay kit (CD4-FITC, CD25-APC) was obtained from BD, USA. An automatic microplate reader was obtained from Thermo Fisher Scientific, USA. An enzyme-linked immunosorbant assay (ELISA) kit (IL-10, HPV) was purchased from R&D Systems, USA.

### Sample collection

Venous blood (3-5 mL) was collected in heparinized anticoagulant tubes from subjects in the control and observation groups. All of the subjects underwent gynecological examinations. Samples were collected by trained clinicians. A small specialized brush was gently rotated 3-5 times in the cervical canal to collect exfoliated cells for HPV testing.

### Flow cytometry

A total of 50 μL of peripheral blood was added to tubes containing 5 μL of the CD4+, CD25+, or CD4+CD25+ antibody mixtures. The tubes were incubated in the dark for 15 min, and then, 200 μL of red blood cell lysis buffer was added. After the reaction was completed, 1 mL of PBS was added, and the tubes were centrifuged. The supernatant was discarded, 0.5 mL of PBS buffer was added, and the samples were immediately used for the flow cytometry assay.

### Enzyme-linked immunosorbent assay

The coating antigen was diluted in the coating buffer to the required concentration of HBsAg, and 200 μL of the solution was added to each well. The samples were incubated overnight at 4°C and then washed once with double distilled H_2_O. A blocking solution was added to each well. The collected peripheral blood was centrifuged at 3000 r/min prior to addition to the plate. The enzyme-labeled antibody was added, and the solution was mixed prior to sealing the plate. The plate was washed 5 times for 30 s to 1 min each with a prepared cleaning solution. The substrate (100 μL) was added to each well to start the reactions, and the color was monitored. Then, 0.05 mL of 2 M sulfuric acid was added, and the color developed within 15 min.

### Follow-up

Follow-up of the 70 patients was conducted at the following intervals: monthly for 3 months after surgery, every 6 months for the first year, and then annually thereafter. Patients were monitored for a total of 5 years.

### Statistical methods

SPSS 22.0 statistical software (Beijing Strong-Vinda Information Technology Co., Ltd) was used for the statistical analysis of the collected data. GraphPad Prism 5 was used to generate figures with the analyzed data. Survival analysis was performed by the log-rank test. Continuous variables are presented as the mean ± SD. Comparisons between groups were performed with a t test. HPV rates in the two groups were compared using the chi-square test. Comparisons among groups were performed with a single factor analysis. Multivariate correlations were analyzed with Pearson correlation analysis.

## RESULTS

### HPV infection

HPV tests were performed for the 140 subjects. Among the 70 patients with CC, 67 were HPV positive, for an infection rate of 95.7%. Among the 70 subjects in the control group, 8 were HPV positive, for an infection rate of 11.4%. The HPV infection rate differed significantly between the two groups (χ^2^=42.167, *p*<0.05), as shown in Table 2.

### Serum IL-10 levels in CC patients and control subjects

**Figure 1:**
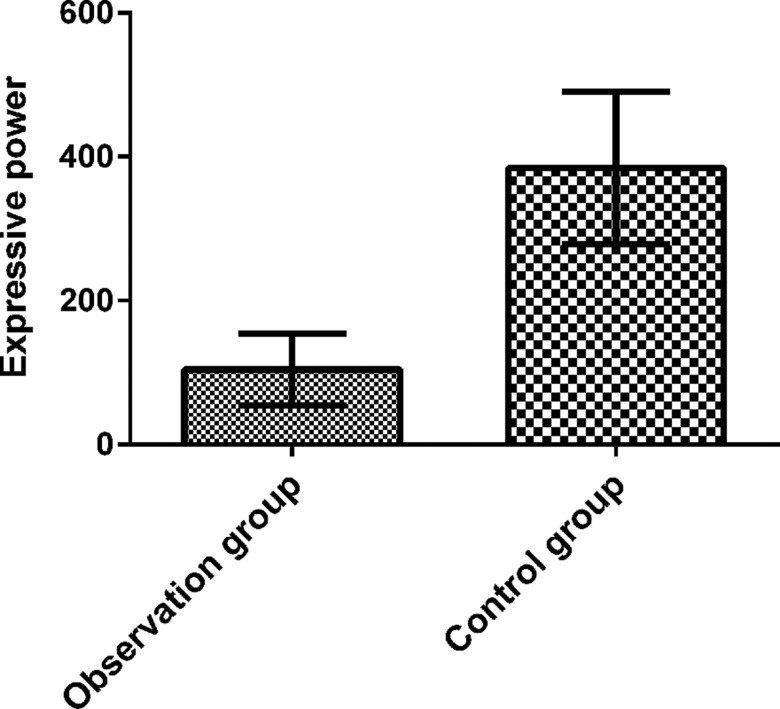
CC patient and control subject serum IL-10 levels. IL-10 levels were higher in CC patients than in control subjects (*p*<0.05).

The serum IL-10 tests revealed serum IL-10 levels of 104±50 pg/mL in the control group and 384±106 pg/mL in the CC group. A significant difference was found between the two groups (*p*<0.05), as shown in Figure 1.

### Peripheral CD4+CD25+ Treg cell ratios

**Figure 2:**
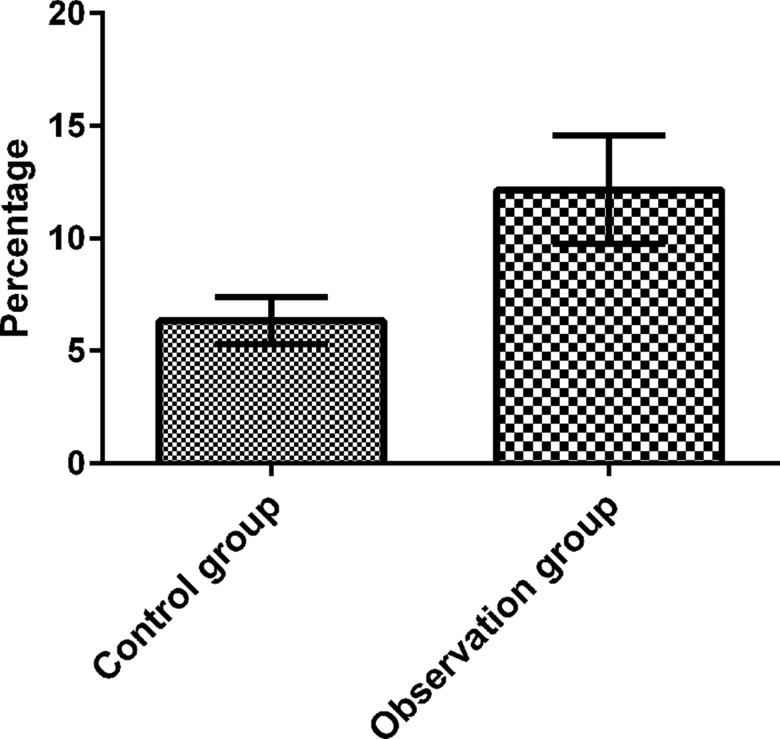
The PBCDT proportions in the observation and control groups. The PBCDT proportion was determined by flow cytometry. The results show that the PBCDT proportion in the peripheral blood was significantly higher in the observation group than in the control group (t=5.684, *p*<0.05).

The peripheral blood CD4+CD25+ Treg cells (PBCDTs) of patients were analyzed by flow cytometry. There was a significant difference in the percentage of PBCDTs in the total CD4+ lymphocyte population between patients with CC (12.16±2.41) and control subjects (6.34±1.05, t=5.684, *p*<0.05), as shown in Figure 2.

### Relationship between the PBCDT percentage and IL-10 in patients with CC

The relationship between the PBCDT percentage and serum IL-10 levels in the 70 patients with CC was determined. The PBCDT percentage was positively correlated with IL-10 expression (r=0.375, *p*<0.05). IL-10 levels increased with rising PBCDT levels.

### Follow-up

**Figure 3:**
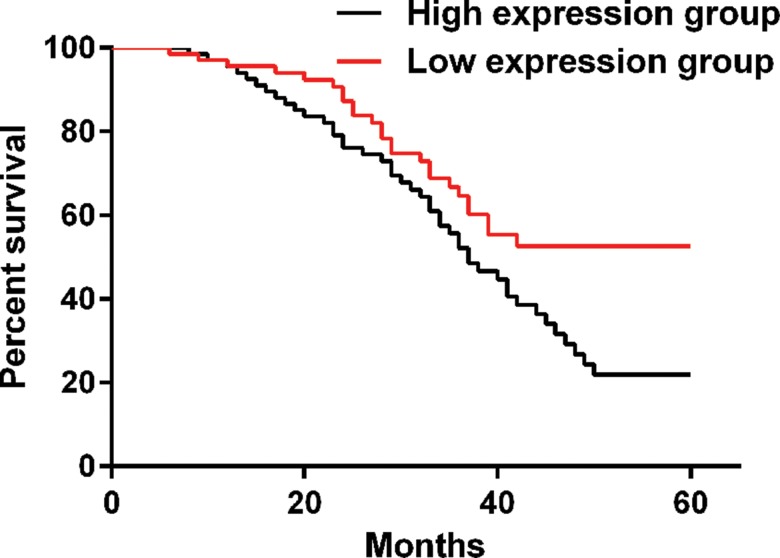
Kaplan-Meier curve. The Kaplan-Meier curve shows that the 5-year patient survival rate was 64.2% in the low IL-10 group and 42.8% in the high IL-10 group. A significant difference was observed between the two groups (*p*=0.012).

Patients were divided into a high IL-10 group and a low IL-10 group according to the median IL-10 level for follow-up. The 5-year patient survival rate was 64.2% in the low IL-10 group and 42.8% in the high IL-10 group. A significant difference was observed between the two groups (*p*=0.012), as shown in Figure 3.

## DISCUSSION

PBCDTs are a class of negatively regulated immune cells that are characteristically identified by the surface expression of CD4 and CD25. T cells that are positive for CD4 and CD25 are mainly produced in the thymus and may also be produced during an immune response to a peripheral tolerogen through induction by TGF-β, αβ T cell receptor signals, and cytokines. CD4 and CD25 play a crucial role in immune control 15-17. It has been reported 18,19 that PBCDTs play an important role in the tumor microenvironment, where they inhibit the T cell population involved in the immune response by affecting cellular activity, thereby exerting important immunosuppressive function. Treg cells are important members of the CD4+ T lymphocyte population in the human body; they can regulate immune tolerance by inhibiting the activation of the immune system. A previous article 18 proposed that Treg cells can dynamically regulate the immune system to achieve balance in healthy humans, thereby inhibiting the expansion of the immune response and the development of autoimmunity.

A previous study proposed that Treg cells can be divided into intrinsic Treg cells and induced Treg cells 20. Intrinsic Treg cells exert inhibitory effects through direct contact, while induced Treg cells have inhibitory effects through the secretion of inhibitory factors, such as IL-10 and TGFβ. IL-10 is an important immunoregulatory factor with inhibitory effects in a variety of cells; its main function is to prevent tissue damage caused by specific and nonspecific immune responses. IL-10 is mainly produced following the induction of IL-10 transcription, synthesis, and secretion by bacterial lipopolysaccharide and catecholamines, but it has been reported previously that Treg cells can also produce IL-10 21, and other studies have found that Th17 and Th22 cells produce IL-10 22,23.

In this study, the HPV-positive rate, the PBCDT count, and serum IL-10 levels were examined in 140 subjects. The HPV-positive rate was higher in the observation group than in the control group, demonstrating that HPV plays an important role in the pathogenesis of CC. A higher HPV viral load is associated with a higher probability of the progression of cervical intraepithelial neoplasia lesions to CC 24. The PBCDT count was higher in the observation group than in the control group. It has been reported that an increase in the human Treg cell count inhibits CD4+ T cell activation and proliferation and cytokine secretion during tumorigenesis, resulting in the generation of an immune response; these data indicate that elevated PBCDT populations can be used as a diagnostic indicator in CC 25. An examination of serum IL-10 levels revealed a significant difference between the control group and the observation group, suggesting that IL-10 is an important negative regulator of the tumor immune microenvironment and can promote tumor growth and metastasis. Our correlation analysis revealed a positive correlation between the PBCDT percentage and IL-10, which proved that Treg cells can inhibit tumorigenesis through the secretion of inhibitory factors. In the last stage of the study, patients were followed up for five years and stratified according to their serum IL-10 levels. The five-year survival rate of the patients with high IL-10 expression was lower than that of those with low IL-10 expression. High tumor IL-10 expression can be used as a predictor of tumor stage. In recent years, an increasing number of reports have demonstrated that Treg cells are involved in the formation of the tumor microenvironment in CC patients and in the development and metastasis of CC, which proves that Treg cells can be used for the clinical diagnosis of CC.

There are some limitations of this study. This study did not include an in-depth analysis of the mechanisms of IL-10 and Treg cells in CC. It is unknown whether the small sample size and regional differences of the patients introduced bias into our results. In future studies, we will increase the sample size and investigate the mechanisms underlying the roles of IL-10 and Treg cells in CC, thereby providing new methods and ideas for the development of antitumor immunotherapy.

In summary, this study demonstrated that the PBCDT ratio and serum IL-10 levels are related to CC activity. These factors mutually influence each other and are involved in the development and progression of CC.

## AUTHOR CONTRIBUTIONS

Wang B and Wang L conceived the study and designed the experiments. Wang H, Li P and Wang LL contributed to the data collection. Liu H and Liu J performed the data analysis and interpreted the results. Wang B wrote the manuscript. Wang L contributed to the critical revision of article. All authors read and approved the final version of the manuscript.

## Figures and Tables

**Table 1:** Patient clinical information.

Group	n	Observation group	Control group	F	*p*
Age					
>55	79	44 (55.69)	35 (44.31)	0.511	0.695
≤55	61	26 (42.62)	35 (57.83)		
Clinical stage					
I		9 (12.85)			
II		45 (64.28)			
III		16 (22.87)			
Pathological classification					
Adenocarcinoma	3 (4.28)				
Squamous cell carcinoma	66 (94.28)				
Squamous adenocarcinoma	1 (1.44)				
Smoking history					
Yes	44	24 (54.54)	20 (45.46)	1.431	0.084
No	96	59 (61.45)	37 (38.55)		
Exercise habit					
Yes	80	44 (55.00)	36 (45.00)	0.775	0.354
No	60	29 (48.33)	31 (51.67)		
Home area					
Urban	88	51 (57.95)	37 (42.05)	0.947	0.284
Rural	52	29 (55.76)	23 (44.23)		
Degree of education					
<High school	100	50 (50.00)	50 (50.00)	0.797	0.387
≥High school	40	24 (60.00)	16 (40.00)		
Marital status					
Married	131	66 (50.39)	65 (49.61)	0.647	0.667
Unmarried	6	3 (50.00)	3 (50.00)		
Widowed	3	2 (66.67)	1 (33.33)		
Dietary preference					
Mild	85	47 (55.29)	38 (44.71)	0.856	0.394
Spicy	55	29 (47.27)	26 (52.73)		

**Table 2:** HPV infection.

Group	n	HPV infection		Positive rate (%)	χ^2^	*p*
		Positive	Negative			
Observation group	70	67	3	95.7%	42.167	<0.05
Control group	70	8	62	11.4%		
